# The Nutritional Potential of Avocado By-Products: A Focus on Fatty Acid Content and Drying Processes

**DOI:** 10.3390/foods13132003

**Published:** 2024-06-25

**Authors:** Roko Marović, Marija Badanjak Sabolović, Mladen Brnčić, Antonela Ninčević Grassino, Kristina Kljak, Sandra Voća, Sven Karlović, Suzana Rimac Brnčić

**Affiliations:** 1 Faculty of Food Technology and Biotechnology, University of Zagreb, Pierottijeva 6, 10000 Zagreb, Croatia; rmarovic@pbf.hr (R.M.); mbadanjak@pbf.hr (M.B.S.); aninc@pbf.hr (A.N.G.); skarlovi@pbf.hr (S.K.); srimac@pbf.hr (S.R.B.); 2 Faculty of Agriculture, University of Zagreb, Svetošimunska cesta 25, 10000 Zagreb, Croatia; kkljak@agr.hr (K.K.); svoca@agr.hr (S.V.)

**Keywords:** avocado, by-product, hot-air microwave drying, nutritional value, oleic acid, tocopherols

## Abstract

The aim of this study was to analyze the content of fatty acids and tocopherols in various components (pulp, seeds, peel) of avocado (*Persea americana*), which are often neglected as by-products. In addition, the effects of different drying processes on these components were investigated and the health benefits of the main fatty acids contained in avocados were highlighted. The samples were subjected to three drying processes: hot air (HAD), vacuum (VD), and hot-air microwave (HAMD). In all parts of fresh avocado, oleic acid was the most abundant (41.28–57.93%), followed by palmitic acid (19.90–29.45%) and linoleic acid (8.44–14.95%). Drying led to a significant reduction in the oleic acid content, with palmitic acid showing the greatest stability. HAD resulted in higher levels of oleic acid and linoleic acid in dried pulp and peel samples compared with VD and HAMD, while HAMD had the highest content of α-linolenic acid in all parts. In addition, HAMD had the shortest drying time. HAMD duration was 35 min, which was 76.7% shorter than HAD (150 min) and 82.5% shorter than VD (200 min). Considering fatty acid retention and drying efficiency, HAMD appears to have been the most effective method, especially for the avocado peel. Remarkably, the avocado peel consistently contained higher total tocopherol, with δ-tocopherol generally being the most abundant form. The high content of tocopherols, oleic acid, and linoleic acid in the avocado peel suggests promising health benefits.

## 1. Introduction

Avocado (*Persea americana*) is a fruit that originates from the subtropical and tropical regions of Mexico and Central America. It has been a staple food for over 9000 years in these regions [1]. In 2019, global avocado production amounted to 7.31 million tons, with Mexico accounting for the largest share at 31.5%, followed by significant contributions from the Dominican Republic (9.1%), Peru (7.3%), and Colombia (7.3%) [2,3]. Recent data from FAOSTAT [4] indicates a further increase in production to 8.98 million tons.

Due to its exceptional nutritional profile and well-established health benefits, avocado is often referred to as a ‘superfood’. Avocado has a high content of soluble and insoluble fiber, beneficial fats, and a range of essential minerals (such as magnesium, potassium, copper, phosphorus, and zinc) and vitamins (including vitamin E, vitamin C, vitamin K, and various B-group vitamins) and also contains a wealth of bioactive compounds such as carotenoids, phenols, phytoestrogens, acetogenins, and tannins. This comprehensive nutrient profile contributes to numerous overall positive effects on human health through the action of antioxidants, anti-inflammatory agents, cardioprotective substances, and antimicrobial agents with potential anti-cancer properties. For example, carotenoids, tocopherols, vitamin C, and phenolic compounds serve as antioxidants and neutralize oxidative stress, helping the body maintain homeostasis. In addition, the combination of fiber, oleic acid, linoleic acid, potassium, and vitamin B6 contained in avocados can act synergistically to reduce the risk of cardiovascular disease [5,6,7].

The avocado fruit consists of three main components: pulp, peel, and seed. Both direct consumption and industrial processing of avocados generate considerable residues, with peels and seeds accounting for about 11% and 16% of the total weight of the fruit, respectively [8]. While the pulp is commonly consumed in households and restaurants, the extraction of avocado oil generates significant amounts of pulp residue, which has considerable nutritional value and is therefore treated as a by-product [9]. Despite the substantial production volumes, the inadequate and insufficient utilization of avocado residue represents a significant environmental challenge. Currently, most of this food by-product is discarded or not fully utilized [10]. However, avocado by-products hold immense potential for various industrial applications, including the food industry, agro-processing industry, pharmaceutical industry, cosmetic industry, textile industry, biopolymer industry, etc. [11].

Due to their pronounced climacteric nature and high fat content, avocados and their by-products are more susceptible to spoilage compared with other fruits, resulting in a significantly shorter shelf life. Therefore, there is an urgent need for research into effective drying methods to extend shelf life, improve stability, and enable various applications for avocados [12]. Drying is used as a preservation method for fruits and vegetables to extend shelf life while maintaining product quality and stability. This method involves the reduction of water activity and moisture content, critical factors in mitigating microbiological contamination during storage and preventing possible deterioration of the product [13,14]. There is a plethora of drying techniques for various raw materials, each tailored to produce high-quality products with a longer shelf life. Vacuum drying (VD) uses reduced pressure and lower temperatures, which helps to preserve heat-sensitive nutrients and the sensory properties of food. This method offers several advantages for heat-sensitive materials that can decompose under atmospheric pressure and higher temperatures. In VD, heat transfer occurs primarily via radiation from infrared heaters and via conduction, where heat is transferred through the heated surface, and drying is limited by the surface area available for heat transfer [15]. In hot-air drying (HAD), the water is removed using a stream of hot air, and the water evaporates via simultaneous heat, mass, and momentum transfer. The heat energy is transferred to the material surface through convection and then diffuses or convects inside the material, depending on its structure [16]. HAD is widely preferred in the industry due to its simplicity, ease, and reliability [17]. However, its disadvantages, such as long drying times and slow material heating, can be mitigated through combining it with other methods or applying different pre-treatments to increase efficiency [18]. HAD can be combined with dielectric heating to overcome its inherent limitations. This hybrid approach mitigates issues such as increased diffusion rates and ensuring adequate surface moisture, resulting in shorter drying times and minimized surface crust formation, thereby improving product quality and energy efficiency [19]. Hot-air microwave drying (HAMD) is an energy-efficient process that utilizes the polar properties of water molecules to absorb microwave energy to increase their kinetic energy, thereby generating heat that accelerates the drying rate compared with conventional methods [20].

The aim of this study was to analyze the fatty acid and tocopherol composition of the various parts of Hass avocados: pulp, seed, peel, which are often considered as by-products. In addition, the effects of different drying processes (HAD, VD, HAMD) on these components were investigated. The main focus was on the comparison of HAMD, a novel drying method, with conventional drying methods such as HAD and VD. This comparison focused in particular on the retention of fatty acids and tocopherols in the dried samples and the efficiency of the process in recovering avocado by-products. Based on the fatty acid profiles obtained, this study aimed to provide information on the effects and potential health benefits of the individual avocado parts.

## 2. Materials and Methods

### 2.1. Materials

#### Sample Preparation

Avocado fruits of the Hass variety originating in Peru were purchased from OPG Šulog (Donja Bistra, Croatia). Prior to analysis, the avocados were stored in the dark at a low temperature of 4 °C. Ripe avocados were manually cut and separated into peel, pulp, and seeds. The seeds were crushed using a chopping device, while the peels were manually cut into smaller pieces of about 3–4 cm in length and 2–3 cm in width. The separated avocado parts were then divided into four groups and subjected to different drying processes, resulting in the following sample groups: fresh, HAD, VD, and HAMD. Each drying experiment was performed in triplicate and employed 100 g of fresh sample, which included peel from 5 avocados, pulp from 5 avocados, and seeds from 5 avocados.

### 2.2. Methods

#### 2.2.1. Drying Processes

The prepared samples were subjected to various drying methods. For HAMD, a hybrid hot-air microwave oven/dryer (Estherm, Novaki, Croatia) was used, operating with a power of 360 W, a frequency of 2450 Hz, a temperature of 60 °C, and an airflow of 1.5 m/s. Approximately 100 g of the raw material (sample) was evenly distributed on a stationary ceramic plate. For HAD, the same device was used with a temperature of 60 °C and an airflow of 1.5 m/s. For VD, the samples were dried in a conduction vacuum dryer (VO200 PM200, Memmert GmbH + Co., KG, Schwabach, Germany) at 60 °C temperature and 100 mbar pressure. Approximately, 100 g of the sample was distributed on three stainless steel trays (32–34 g each). Water loss during drying was monitored every 20 min using a laboratory balance (Mettler Toledo ME1002TE, Columbus, OH, USA). All drying procedures were continued until a consistent mass was achieved. The dried samples were then ground into powder using an electric grinder. Subsequently, all dried samples were vacuum sealed and stored in a dark environment until further analysis.

#### 2.2.2. Fatty Acids Determination

The total fat content was extracted according to the protocols of the Association of Official Analytical Chemists (AOAC) [21]. Gravimetric determination of total fat content was conducted with petroleum ether for 8 h in a Soxhlet apparatus. The resulting extracts were then used for fatty acid analysis. The fatty acid methyl esters were prepared following the guidelines of the ISO 15884 standard [22]. Extracted fat weighing 100 mg was measured in microreaction vials and then, 5 mL of hexane and 0.2 mL of transesterification reagent (2 mol/L sodium methoxide in methanol) were added. The mixtures were stirred vigorously on a vibrating mixer for 1 min and allowed to settle for 5 min. Subsequently, 0.5 g of NaHSO_4_ × 4H_2_O was added and the mixtures were stirred again before centrifugation at 350× *g* for 3 min at room temperature. The clear supernatants were then transferred to vials. The method for fatty acids determination was created using default parameters: (i) injector temperature: 250 °C; (ii) column temperature: 50 °C/5 min → 5 °C/1 min → 260 °C/30 min; (iii) carrier gas: helium; (iv) injection volume: 1 µL; (v) split ratio: 1:50. The identification of the fatty acids was performed via comparing the retention times (RT) of the recorded chromatograms of the samples and the standard FAME Mix 37. The prepared samples were analyzed according to the ISO 15885 standard [23] on a gas chromatograph (GC) instrument (Shimadzu, Japan). An InertCap Pure Wax column (0.25 mm I.D. × 30 m, df = 0.25 μm, Shimadzu, Japan) and a flame ionization detector (FID) were used for fatty acid analysis. The results of the areas of all marked peaks on the chromatogram were visualized using Automatic Program Software Solution GC, Version 2.41.00 (Shimadzu, Japan). The quantification of the proportions of the individual fatty acids in the sample was carried out via comparing the sample with the FAME Mix 37 standard (CRM47885, Sigma-Aldrich, St. Louis, MO, USA).

#### 2.2.3. Tocopherols Determination

Tocopherols were extracted using the method described by Montoya-Arroyo et al. [24]. Dry samples were pulverized using a ball grinder (MM 200, Retsch, Germany) and, in triplicate, 100 mg of each sample was mixed with 900 mL of ultrapure water and ultrasonicated (10 min; Sonorex TK 52, Bandelin, Berlin, Germany). Then, 2 mL of ethanol containing 1% of ascorbic acid and 60 µL of saturated KOH was added and mixtures were saponified for 30 min at 70 °C under continuous agitation. After incubation, mixtures were cooled on ice and lipophilic compounds were extracted with 2 mL of hexane (HPLC grade). After centrifugation (10 min at 2200× *g*; Centric 322A, Tehtnica, Železniki, Slovenia), the hexane layer was separated and the extraction procedure was repeated until a colorless upper layer was present. The collected supernatants were evaporated using a rotary vacuum concentrator (RVC 2-25CD plus, Martin Christ, Osterode am Harz, Germany). The residue was dissolved in 200 μL of acetonitrile–dichloromethane–methanol (45:20:35, *v*/*v*/*v*) containing 0.1% BHT. Extractions were carried out under dim light, and the extracts were analyzed further using high-performance liquid chromatography (HPLC) on the same day. Tocopherols were separated and quantified using a SpectraSystemHPLC instrument (Thermo Separation Products, Inc., Waltham, MA, USA) equipped with a quaternary gradient pump (P4000), an autosampler (AS3000), a UV-Vis detector (UV2000), and an FL detector (FL3000). Compounds were separated on two sequentially connected C18 reversed-phase columns [25,26]: a Vydac 201TP54 column (5 μm, 4.6 mm × 150 mm; Hichrom, Reading, UK) followed by a Zorbax RX-C18 column (5 μm, 4.6 mm × 150 mm; Agilent Technologies, Santa Clara, CA, USA). The separation columns were protected with a Supelguard Discovery C18 guard column (5 μm, 4 mm × 20 mm; Supelco, Bellefonte, PA, USA). The mobile phase consisted of acetonitrile–methanol–dichloromethane (75:25:5, *v*/*v*/*v*) containing 0.1% BHT and 0.05% triethylamine. An aliquot of 30 μL was injected, and the flow rate was 1.8 mL/min. The separations were performed at room temperature. Tocopherols were monitored at an extinction of 290 nm and an emission of 330 nm. They were identified via comparing their retention times and quantified using external standardization with calibration curves, using commercially available standards (R^2^ ≥ 0.99). Tocol standards (α-, γ-, and δ-tocopherol; purity ≥ 93%) were obtained from Supelco (Sigma-Aldrich, St. Louis, Missouri, USA).

Vitamin E activity, expressed as mg of tocopherol equivalent per kg of oil, was calculated according to Equation (1) [27]:

Vitamin E activity = α-tocopherol + 0.4 × β-tocopherol + 0.1 × γ-tocopherol + 0.01 × δ-tocopherol(1)

#### 2.2.4. Fatty Acid Profile Functionality Evaluation

The functionality of the fatty acid composition of different avocado parts was determined through calculating four different indices. For calculating these indices, the amounts of saturated fatty acids (SFA), monounsaturated fatty acids (MUFA), polyunsaturated fatty acids (PUFA), and total unsaturated fatty acids (TUFA) in avocado parts were used. PUFA–SFA ratio is the most commonly used index for assessing the nutritional and health value of fat content in food sources, and was calculated according to Equation (2) [28]. TUFA–SFA ratio takes into consideration MUFA, being a more precise predictor of the influence of fatty acid profile on cardiovascular health. It was calculated according to Equation (3) [29]. Index of atherogenicity (IA) was calculated according to Equation (4) [30], and hypocholesterolemic to hypercholesterolemic ratio (H/H) was calculated according to Equation (5) [31]:

(2) $$\mathrm{PUFA}\text{/}\mathrm{SFA}=\frac{\Sigma \;PUFA}{\Sigma \;SFA}$$

(3) $$\mathrm{TUFA}\text{/}\mathrm{SFA}=\frac{\left(\Sigma \;MUFA+\Sigma \;PUFA\right)}{\Sigma \;SFA}$$

(4) $$\mathrm{IA}=\frac{\left(C12\text{:}0+4\times C14\text{:}0+C16\text{:}0\right)}{\left[\Sigma \;MUFA+\Sigma \;PUFA\;(C18\text{:}2+C18\text{:}3)\right]}$$

(5) $$H\text{/}H=\frac{\left(C18\text{:}1+C18\text{:}2+C18\text{:}3\right)}{\left(C12\text{:}0+C14\text{:}0+C16\text{:}0\right)}$$

where C12:0 is lauric acid, C14:0 is myristic acid, C16:0 is palmitic acid, C18:0 is stearic acid, C18:1 is oleic acid, C18:2 is linoleic acid, C18:3 is α-linolenic acid, ∑ SFA is the sum of saturated fatty acids, ∑ PUFA is the sum of polyunsaturated fatty acids, and ∑ MUFA is the sum of monounsaturated fatty acids. TUFA is the sum of MUFAs and PUFAs.

### 2.3. Statistical Analysis

The results were expressed as mean ± standard deviation of triplicate measurements (*n* = 3). Significant differences (*p* < 0.05) between samples within means were examined using analysis of variance (ANOVA) and Tukey’s test for honestly significant differences (HSD). All tests for data analysis were evaluated using SPSS Statistics software version 26 (IBM, New York, NY, USA).

## 3. Results and Discussion

### 3.1. Fatty Acid Composition in Fresh Avocado Parts

As shown in Table 1, oleic acid (C18:1) was the predominant fatty acid in all fresh avocado parts (pulp, seed, peel), indicating its significance in avocado composition. Oleic acid (C18:1) served as the main representative of monounsaturated fatty acids (MUFAs) in fresh avocado parts. The pulp (57.93%) and peel (56.79%) contained higher concentrations of oleic acid compared with the seed (41.28%). Palmitic acid (C16:0), the second most abundant fatty acid and the most important representative of the saturated fatty acids (SFAs), had the highest content in the seed (29.45%), followed by the pulp and peel (20.34% and 19.90%, respectively). Palmitoleic fatty acid (C16:1), which serves as a secondary MUFA representative, showed relatively constant contents in the different fresh avocado parts. Among the polyunsaturated fatty acids (PUFA), linoleic acid (C18:2) was the most abundant with values of 14.95%, 11.62%, and 8.44% in seed, peel, and pulp, respectively. In addition, rare long-chain fatty acids such as behenic acid (C22:0) and nervonic acid (C24:1) were also detected.

In the mesocarp (pulp) of the avocado, oleic acid (C18:1) was identified as the most abundant fatty acid, followed by palmitic acid (C16:0), linoleic acid (C18:2), and palmitoleic acid (C16:1). These results were consistent with those of Opiyo et al. [32], who analyzed the pulp of fourteen Hass avocados of different origins. In another study, Galvao et al. [33] found the same order of fatty acid content in pulp of the Fortuna and Barker avocado varieties, although palmitic acid predominated in the Collinson variety. Goncalves et al. [34], who analyzed four avocado varieties from Madeira Island, observed that oleic acid (C18:1) consistently ranked highest, followed by linoleic acid (C18:2), with palmitic (C16:0) or stearic acid (C18:0) in third place. In the current study, in the avocado seeds, oleic acid (C18:1), linoleic acid (C18:2), palmitic acid (C16:0), and palmitoleic acid (C16:1) were the most abundant fatty acids. In the fresh peel samples, oleic acid (C18:1) was the most abundant fatty acid at 56.79%. This observation was consistent with previous studies: for fresh peels, Ramos-Aguilar et al. [35] reported oleic acid (C18:1) content between 45.56% and 56.84%, while Babiker et al. [36] and Al-Juhaimi et al. [37] found oleic acid (C18:1) content of 56.40% and 59.78%, respectively.

### 3.2. Fatty Acid Composition in Dried Pulp

The fatty acid compositions of the differently dried (HAD, VD, HAMD) pulp samples are listed in Table 2. The predominant fatty acids were oleic acid (C18:1), palmitic acid (C16:0), linoleic acid (C18:2), and palmitoleic acid (C16:1). After the drying processes, an oleic acid (C18:1) content of 44.42% was found in the HAD pulp, 42.81% in the VD pulp, and 44.01% in the HAMD pulp. The palmitic acid (C16:0) content in HAD, VD, and HAMD was found to be 26.15%, 30.18%, and 26.01% respectively. Minimal variation was observed in the palmitoleic acid (C16:1) content, with percentages of 12.23% (HAD), 12.11% (VD), and 13.02% (HAMD). The linoleic acid content ranged from 10.19% in the VD sample to 15.44% in the HAD sample.

In summary, these results showed that, compared with the oleic acid (C18:1) content of fresh samples (Table 1), specific drying processes led to a partial reduction of the oleic acid content in the avocado pulp. This reduction led to an increase in the content of other dominant fatty acids (palmitic acid (16:0), linoleic acid (18:2), palmitoleic acid (C16:1)) without necessarily increasing their absolute amounts. The apparent decrease in oleic acid content can be attributed to the heat generated during drying, which could cause oxidation of the double bond and facilitate the degradation of oleic acid. The lowest levels of oleic acid and linoleic acid were observed in VD pulp, resulting in the lowest TUFA content among all drying treatments. This indicates that oxidation reactions proceeded at a higher rate during VD, resulting in the formation of more degradation products. Chimsook and Assawarachan [38] investigated the effects of different drying methods on avocado pulp and found that VD resulted in higher acid values and peroxide number values compared with HAD. In contrast, Al-Juhaimi et al. [37] reported no significant changes in the fatty acid composition of the pulp of the Fuerte variety after drying processes (HAD, air, microwave). Moreover, Babiker et al. [36], who examined the pulp of the Pinkerton cultivar, also observed an increase in the oleic acid content after treatment with all drying systems (HAD, air, microwave). Krumreich et al. [39] observed that HAD or VD at 60 °C compared with HAD at 40 °C resulted in lower peroxide numbers and iodine values, suggesting that too low temperatures may not have effectively inactivated the enzymes. These different findings might have been due to the differences in the avocado cultivars studied and the different drying methods and conditions that were applied.

### 3.3. Fatty Acid Composition in Dried Seed

In Table 3, fatty acid compositions of the dried seed samples are shown. The most abundant fatty acids determined were oleic acid (C18:1), linoleic acid (C18:2), palmitic acid (C16:0), and palmitoleic acid (C18:2). Compared with the fatty acid composition in other parts (pulp, peel) of the avocado fruit, the fatty acid content in the seeds varied significantly more. Specifically, linoleic acid (C18:2) had the highest values in the HAD seed with 42.45%, followed by 39.83% in the VD seed, and 13.67% in the HAMD sample. Oleic acid (C18:1) was determined in HAD, VD, and HAMD samples with percentages of 34.42%, 33.56%, and 42.33%, respectively. Palmitic acid (C16:0) was determined in HAD, VD, and HAMD with 29.45%, 15.19%, 19.27%, and 24.77%, respectively. Palmitoleic acid (C18:2) in the seed was obtained at lower proportions compared with the other avocado parts, reaching its maximum value in an HAMD sample at 11.06% and decreasing to its minimum value in an HAD seed at 3.87%.

When considering all parts of the avocado, the seed may be less resistant to oxidation and changes in ambient conditions in terms of fatty acid stability, as it has a higher initial content of PUFAs (mainly linoleic acid) than the other parts of the avocado. Changes in linoleic acid (C18:2) can occur when the seeds are exposed to oxygen and heat. The degradation of linoleic acid (C18:2) is caused by the oxidation reaction on unsaturated bonds and is characterized by the accumulation of secondary products such as aldehydes or ketones. The VD sample might be considered the most representative sample of the original fatty acid composition in avocado seed, considering that the results of this study are in agreement with those of Morais et al. [40], who determined that linoleic acid (C18:2) is the main fatty acid in fresh avocado seed. Also, in agreement with these results, Galvao et al. [33] reported that linoleic acid was the most abundant fatty acid in three different avocado cultivars (Baker, Collinson, Fortuna), ranging from 23.95% to 29.38%.

### 3.4. Fatty Acid Composition in Dried Peel

The fatty acid composition of dried peel samples is presented in Table 4. When analyzing the fatty acid composition in differently dried (HAD, VD, HAMD) Hass cultivar avocado peel samples, seventeen different fatty acids were identified. Among them, oleic acid (C18:1), palmitic acid (C16:0), linoleic acid (C18:2), and palmitoleic acid (C16:1) were the most abundant. The oleic acid (C18:1) content was measured at 47.47%, 44.53%, and 41.71% in the HAD, VD, and HAMD samples, respectively. Palmitic acid (C16:0) was the second most abundant fatty acid in all samples, with contents ranging from 21.78% (HAD) to 25.59% (VD), which was consistent with findings reported by other authors. For instance, Ramos-Aguilar et al. [35] and Babiker et al. [36] reported ranges for palmitic acid (C16:0) from 16.69% to 25.02% and 15.77% to 20.82%, respectively.

These slight variations in palmitic acid (C16:0) content were largely predictable due to its chemical structure and properties. Namely, palmitic acid (C16:0), which is classified as a saturated fatty acid (SFA), has a higher resistance to oxidation, which makes it more stable when dried and exposed to heat. While the absolute amounts of palmitic acid (C16:0) remained constant during drying, the relative increase in its proportion in the dried samples compared with the fresh samples (Table 1) could have been due to a decrease in the amount of oleic acid (C18:1). Regarding linoleic acid (C18:2), its content was measured at 11.62% in the fresh sample (Table 1). In all dried samples, the percentage of linoleic acid in the total fatty acid composition increased and ranged between 16.12% and 17.99%. These results are in agreement with those of Babiker et al. [36], who studied Pinkerton avocado peel and reported an increase in linoleic acid (C18:2) from 13.54% in the fresh sample to 18.2% with microwave drying and 19.80% with HAD drying. In contrast, Al-Juhaimi et al. [37] observed no significant changes in linoleic acid (C18:2) content after drying avocado peel of the Fuerte cultivar. It cannot be concluded with certainty that the proportional increase in linoleic acid (C18:2) was solely due to the decrease in oleic acid (C18:1). For example, Morais et al. [40] determined an increase in the mass concentration of linoleic acid (C18:2) in avocado peel through comparing the amount in fresh peel (0.466 g/100 g dry matter) with that in oven-dried peel (0.613 g/100 g dry matter). Babiker et al. [36] suggested that the increase in linoleic acid (C18:2) after drying treatment was likely to have been due to the thermal degradation of the cell matrix and the decomposition of conjugated lipids and phospholipids, thereby releasing more free forms of linoleic acid (C18:2). It seems that microwave drying had a slightly more pronounced impact on oleic acid concentration compared with other drying methods, resulting in a higher degradation rate, as reflected in the lowest content of oleic acid in HAMD. In contrast, Al-Juhaimi et al. [37] observed an increase in oleic acid (C18:1) content after oven and microwave drying, with values of 63.59% and 68.91%, respectively, while Babiker et al. [36] reported no significant change in oleic acid (C18:1) content following oven and microwave drying. These relatively contradictory findings and the contrasting effects of the drying methods on the oleic acid (C18:1) content compared with the results of other studies can be attributed to the different drying conditions and the specific avocado cultivars examined. However, the findings of Al Juhaimi et al. [41] support the results of this study; they observed that the application of microwaves (360 W, 540 W, and 720 W) for 5 min caused a decrease in oleic acid (C18:1) in apricot seed oil. In agreement with this, Caponio et al. [42] reported that microwave treatment (1100 W, 15 min) lowered the oleic acid (C18:1) content in olive oil and peanut oil. The same authors concluded that microwave heating at higher power increased the oxidation rate of unsaturated fatty acids, leading to the formation of degradation products that were evaluated as non-eluted material or other components. Moreover, microwave treatment of peanut oil led to the formation of trans isomers, which are harmful to human health. Chiavaro et al. [43] investigated the influence of microwave heating with a power of 720 W on rapeseed and peanut oil at three different times. After 3 min of treatment, the peroxide number increased, indicating that oxidation had taken place; after 6 min, secondary products (aldehydes, ketones) of oxidation were detected, and after 15 min of treatment, free fatty acids were detected, indicating that a hydrolysis process had also taken place. Dostalova et al. [44] heated rapeseed and sunflower oil at a power of 500 W and after 9 min, the peroxide value in both oils increased more than 10-fold. The same authors concluded that the hydroperoxides formed in the initial phase of oxidation can convert into conjugated dienes, which might be even better indicators of the oxidation-induced degradation of fatty acids. However, Pop [45] could not detect any changes in rapeseed oil after treatment with 900 W for 5 min. Interestingly, when looking at the total amount of unsaturated fatty acids (TUFAs) found in this study, based on the results in Table 2, VD showed the lowest content, which was due to the lowest levels of PUFAs, linoleic and linolenic fatty acids among all the drying treatments.

### 3.5. Fatty Acid Composition of Avocado and Health

Fatty acids, derived from dietary fats, are vital components of cell membranes and energy sources. After digestion and absorption, they are distributed into cells, where they are incorporated into the cell membranes or serve as energy substrates [28]. Fatty acids play an important role in the phospholipids of cell membranes and contribute to certain functions, metabolism, and signaling pathways. Different fatty acid compositions in different cells influence the membrane fluidity, flexibility, and function of membrane proteins [46]. While most of the required fatty acids can be synthesized endogenously by humans, there are exceptions such as the omega-6 fatty acid linoleic acid (LA) and the omega-3 fatty acid alpha-linolenic acid (ALA) [32].

The pulp of the avocado is characterized by its high lipid content, consisting mainly of oleic, palmitic, linoleic, and palmitoleic acids, which makes it a rich source of lipophilic nutrients and bioactive phytochemicals. These components are dissolved and integrated in the avocado’s fat content [47]. Among these nutrients, vitamin E is particularly abundant. It serves as a powerful antioxidant with various beneficial functions in the human body, including the prevention of oxidative stress, protection of cell membranes, regulation of platelet aggregation, activation of protein kinase C, and stimulation of the immune system [48].

Oleic acid is the most abundant fatty acid in avocado and the main representative of MUFAs. In general, a high intake of MUFAs in the diet is associated with a lower risk of cardiovascular diseases. It is well known that the Mediterranean dietary pattern is associated with a low prevalence of chronic diseases (especially CVD) among its consumers. Recently, several mechanisms via which MUFAs exert cardioprotective effects have been discovered and explained: (1) they reduce unfavorable low-density lipoprotein cholesterol (LDL-C) and increase favorable high-density lipoprotein cholesterol (HDL-C) due to the ability to stimulate acyl-CoA cholesterol acyltransferase in the liver; (2) they induce endogenous oxidation of fatty acids and thermogenesis, consequently increasing the body’s energy expenditure—compared with PUFAs and SFAs, MUFAs have a better effect on energy metabolism and, thus, on body composition and weight maintenance; (3) they cause a stronger postprandial feeling of satiety, which is associated with lower energy intake. It has been suggested that this mechanism is achieved via elevation of oleoylethanolamide (OEA), a metabolic regulator that controls appetite sensation through influencing the metabolic and reward systems [49]. Oleic acid (C18:1) can have anti-inflammatory effects via reducing the expression of TNF-α and IL-6 genes, and it may have anti-cancer effects via inhibiting the overexpression of the oncogene HER2. Furthermore, a high intake of oleic acid (C18:1) from avocado oil has been associated with a lower risk of prostate cancer [2]. Moreover, substituting part of the palmitic acid (C16:0) in the diet with oleic acid (C18:1) can reduce the negative effects of saturated fatty acids on adipose tissue, skeletal muscle, liver, and pancreatic β-cells. Consequently, insulin sensitivity may be improved and the risk of diabetes type 2 reduced. In addition, oleic acid (C18:1) can maintain adequate levels of the AMP-activated protein kinase enzyme and acts in the same mechanistic way as metformin, the most commonly prescribed medication for type 2 diabetes [50].

Palmitic acid (C16:0) was the second most abundant fatty acid in all parts of the fresh Hass cultivar avocado. A higher consumption of saturated fatty acids correlates with a higher plasma LDL-C level, which increases the risk of cardiovascular disease. Moreover, high consumption of dietary saturated fatty acids is associated with increased body weight, insulin resistance, abnormal glycemic response, and the inflammatory capacity of adipose tissue. However, there is still some disagreement in the literature about the effects of SFAs on cardiovascular health. Since there are different types of SFAs with different dietary sources, different effects on human health might be expected. For example, when considering palmitic acid, in contrast to animal sources, plant sources of palmitic acid do not elevate plasma LDL-C levels, which may indicate that other dietary components in the food matrix strongly influence the atherogenic potential of palmitic acid [49].

Among the PUFAs, linoleic acid (LA) was most abundant in all parts of the fresh avocado, and the seed stands out as the richest source of LA. LA is an omega-6 fatty acid and is essential for humans as it cannot be synthesized endogenously. Although it is known for its precursor role in the synthesis of other omega-6 fatty acids, including γ-linolenic acid (GLA), di-homo γ-linolenic acid (DGLA), and arachidonic acid (AA), LA alone can act as an essential constituent of ceramides, the skin cells that form 50% of the skin’s epidermis. AA is the most important omega-6 fatty acid in humans due to its activity as a substrate for bioactive lipid mediators, of which eicosanoids are the most representative. The eicosanoids derived from AA are involved in the regulation of immunity, inflammation, platelet aggregation, hemostasis, thrombosis, and vascular tone [46]. These effects are essential for the maintenance of homeostasis and the general health of the human organism. On the other hand, constantly elevated levels of omega-6 fatty acids combined with low levels of omega-3 fatty acids may promote systematic inflammation. However, a systematic review of randomized controlled intervention studies concluded that there was no evidence regarding adverse effects of omega-6 fatty acids on inflammatory markers [49]. Moreover, available data from epidemiologic and intervention studies suggest that omega-6 fatty acids are associated with lower levels of inflammatory markers. Furthermore, inverse correlations have been found between LA intake and body mass index (BMI), waist, insulin, and triglycerides levels, while the opposite applies to HDL-C levels. Specifically, replacing 5% of dietary energy from SFAs with PUFAs reduced LDL-C up to 10%, resulting in a significant reduction in CVD risk [51]. LA can reduce LDL-C levels in plasma, very probably as a result of the upregulation of hepatic LDL receptor gene expression. For decades, the scientific literature has emphasized the importance of the omega-6–omega-3 ratio. The relationship between omega-6 and omega-3 fatty acids in inflammation and cardiovascular risk is complex. Djuricic and Calder [46] point out that the relationship between omega-6 and omega-3 PUFAs in the context of inflammation is not yet fully understood. Furthermore, the omega-6–omega-3 ratio is not clearly associated with CVD risk [51,52]. In light of these findings, other ratios were assessed in this study (Table 5).

Among these, the hypocholesterolemic–hypercholesterolemic (H/H) ratio seems to be the most relevant. Unlike the PUFA–SFA ratio, the H/H ratio includes oleic acid as the main representative of MUFAs, excludes those SFAs that are missing a hypercholesterolemic effect, and only considers those (C12:0, C14:0, C16:0) that are showing this effect. This ratio may be more accurate than the PUFA–SFA ratio in terms of assuming effects of the fatty acid compositions of some foods on cholesterol levels in plasma. For example, most fish have an H/H ratio between 1.75 and 2.95, while most meat and dairy products range between 1.57 and 2.78 and 0.32 and 1.29, respectively [28]. In this study, among fresh samples (Table 5), the highest H/H ratio and the greatest hypocholesterolemic potential were determined for the peel with a value of 3.55, followed by pulp with 3.26 and seed with 1.93. Dried avocado samples showed varying H/H ratios, suggesting differing hypocholesterolemic potential.

### 3.6. Tocopherol Composition

Three tocopherol forms were determined: α-tocopherol, γ-tocopherol, and δ-tocopherol. The results were expressed as µg/g of dry weight and are presented in Table 6. Among the fresh samples (pulp, seed, peel), the peel had the highest content of each form of tocopherol. The values determined were: 57.57, 43.23, and 82.42 µg/g for α-tocopherol, γ-tocopherol, and δ-tocopherol, respectively. In the pulp, the values were lower: 24.57, 26.26, and 6.57 µg/g for α-tocopherol, γ-tocopherol, and δ-tocopherol, respectively. Only α-tocopherol, with a value of 13.61 µg/g, was detected in seeds. Compared with the pulp and seed, the peel showed significantly higher vitamin E activity.

The tocopherol compositions of the dried peel samples are shown in Table 7, expressed as µg/g of dry weight. In general, δ-tocopherol was the most abundant form of tocopherol. Compared with the fresh peel, the dried peel samples had higher total tocopherol content, ranging from 253.80 to 268.45 µg/g. Among the dried peel samples, different drying methods led to variation in tocopherol content. The VD peel sample had the highest α-tocopherol (108.81 µg/g) and γ-tocopherol (64.80 µg/g) content, while the HAMD peel sample had the highest δ-tocopherol (130.24 µg/g) and the highest total tocopherol (268.45 µg/g) content. Statistically significant differences were found between the tocopherol contents of avocado peels treated with different drying methods (*p* < 0.05). Interestingly, it was calculated that the VD peel sample expressed the greatest vitamin E activity.

**Figure 1:**
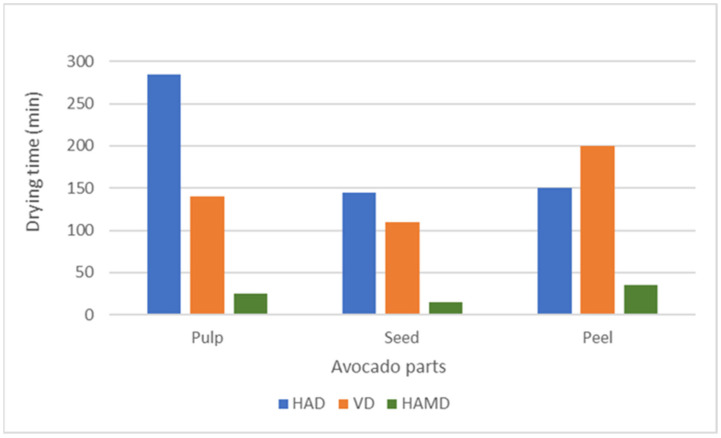
Drying times of different drying processes (HAD, VD, HAMD).

The different drying methods varied in terms of temperature and degree of heat exposure. Heat can break down sensitive compounds such as tocopherols, especially if the temperature is too high or the drying process takes too long. Santana et al. [53] investigated the influence of different drying processes on oil extraction and tocopherol content in peeled and unpeeled pitted avocados and observed that microwave oven drying (912 W, 9 min) showed a higher α-tocopherol content compared with oven drying (60 °C, 5 h), especially for unripe avocados, reaching the maximum value of 21.0 mg/100 g in the oil of the unpeeled samples. In their study, α-tocopherol was the predominant form of tocopherol. Chimssok and Asswarachan [38] determined a higher content of vitamin E in HAD samples (125 mg/kg of oil) compared with VD samples (116 mg/kg of oil). Hu et al. [54] used microwave heating as a pretreatment (700 W, 1–5 min) before oil extraction from peanuts. They noticed an increase in all forms of tocopherols, suggesting that microwaves caused damage to lipoprotein membranes, which could have contributed to the release of tocopherols and improved their content in the extracted oil. However, the mentioned authors warned about the possibility of tocopherol decomposition due to longer microwave application. Ghafoor et al. [55] investigated the tocopherol content in oils extracted from the pulp of three different unripe and ripe avocado varieties (“Hass”, “Fuerte”, and “Pinkerton”) dried under ambient conditions, through microwave treatment (560 W, 15 min), or in the oven (60 °C, 19 h). Their results showed that when fresh Hass avocados were microwave-dried, an increase in γ-tocopherol content was observed, although conversely, α-tocopherol content decreased. This might be due to the structural differences and thus, the different stability and sensitivity of the different types of tocopherols to heat exposure. Exposure to oxygen during the drying process can also affect the tocopherol content. Excessive exposure to oxygen can lead to oxidation of the tocopherols, reducing their concentration. Therefore, the loss of α-tocopherol may be associated with oxidative stress and its protective role in the preservation of fatty acids and phytosterols via scavenging free radicals. In addition, tocopherol content and its stability during drying may vary depending on geographical location, climatic factors, genetic structure, agricultural practices, and the degree of ripeness of the avocado. The duration of the drying process can influence the content of tocopherols and fatty acids. Longer drying times can lead to greater degradation of these compounds, while shorter drying times can preserve them. The various drying methods differ in their efficiency and drying rate, which can affect the preservation of the ingredients. HAD is a common method for drying plant material and is more cost-effective compared with new methods. However, it has significant drawbacks, including drying rate, drying time, energy efficiency, and product quality. The combination of HAD with microwaves can improve the drying process due to the higher heat and mass diffusion caused by volumetric heating [19]. The results of the drying times using different drying methods (HAD, VD, HAMD) for different avocado parts (pulp, seeds, peel) are shown in Figure 1. It was observed that HAMD shortened the drying time compared with VD and HAMD for all parts of the fresh avocado. For pulp drying, HAMD lasted for 15 min, which was 91.2% shorter than HAD (284 min) and 82.2% shorter than VD (140 min). For seed drying, the HAMD drying time was 15 min, which was 89.3% shorter than HAD (140 min) and 87.4% shorter than VD (110 min). For peel drying, the HAMD duration was 35 min, which was 76.7% shorter than HAD (150 min) and 82.5% shorter than VD (200 min).

These results were in agreement with the results of other studies. Alibas and Köksal [56] found that the drying time for microwave drying of mallow leaves was between 6 and 10 min, while the drying times for convective drying (50 °C) and vacuum drying (50 °C, 7 kPa) were 150 min and 130 min, respectively. In another study, the drying of shitake mushrooms with hot-air intermittent microwave drying was 42.86% shorter than infrared drying and 64.29% shorter than hot-air drying [57]. Horuz et al. [58] compared HAMD with HAD and observed a significant reduction in the drying time of sour cherries when HAMD was used. In addition, HAMD reduced the drying time of nectarine slices by up to 95.44% compared with HAD [59]. The different drying rates and times of the HAMD, VD, and HAD drying processes are due to their different thermodynamic properties. In the HAD process, for example, the heat is first transferred from the surrounding hot air to the surface of the material and thus, the temperature gradient runs from the surface to the center. With the HAMD process, the opposite is the case: the temperature gradient runs from the center of the material to the surface. This happens due to the heat generated by the excitation of water molecules caused by the penetration of microwaves into the material.

## 4. Conclusions

All parts of the avocado are rich in oleic acid (C18:1) and serve as important sources of palmitic (16:0), linoleic (C18:2), and palmitoleic (C16:1) acids. Although avocado peels are often discarded, they have a favorable fatty acid profile that may offer health benefits. Considering the hypocholesterolemic properties of oleic (C18:1) and linoleic acids (C18:2), avocado peel may contribute to reducing the risk of cardiovascular disease. In addition, avocado peel is a rich source of tocopherols, various types of vitamin E that are important for human health.

Various drying methods are used to reduce moisture content, increase stability, and prevent spoilage, ultimately aiming to improve the utilization of avocado by-products. In this study, all drying methods (HAD, VD, HAMD) resulted in a significant loss of oleic acid (C18:1) in all avocado parts, while palmitic acid (C16:0) remained relatively stable with minimal variation in content. Among the drying methods, HAD appeared to better preserve the original fatty acid composition of the avocado peel compared with VD and HAMD. However, HAMD proved to be the most efficient process due to its shorter duration, suggesting promising prospects for avocado by-product utilization in terms of sustainability and the green economy. Thus, HAMD represents a promising approach to improving the efficiency and quality of the drying process for avocado by-products, which may have an impact on preserving the fatty acid composition and overall nutritional value of the final product. Nevertheless, further investigation of different drying conditions (microwave power, hot air temperature) within the HAMD process is required to optimize the preservation of the fatty acid composition and to increase the nutritional value of avocado by-products in the future.

## Figures and Tables

**Table 1:** Fatty acid composition (%) in Hass avocado pulp, seed, and peel.

Fatty Acid	Pulp	Seed	Peel
C14:0	-	0.38 ± 0.004 ^A^	0.12 ± 0.002 ^B^
C16:0	20.34 ± 0.22 ^B^	29.45 ± 0.60 ^A^	19.90 ± 0.44 ^B^
C16:1	8.58 ± 0.14 ^A^	8.72 ± 0.33 ^A^	7.60 ± 0.11 ^B^
C17:0	-	-	-
C17:1	-	-	-
C18:0	0.45 ± 0.03 ^B^	-	0.55 ± 0.02 ^A^
C18:1	57.93 ± 0.52 ^A^	41.28 ± 0.67 ^B^	56.79 ± 0.50 ^A^
C18:2	8.44 ± 0.22 ^C^	14.95 ± 0.40 ^A^	11.62 ± 0.34 ^B^
C18:3	-	-	1.33 ± 0.01 ^A^
C20:0	-	1.28 ± 0.13 ^A^	0.13 ± 0.007 ^B^
C20:1	-	-	0.21 ± 0.01 ^A^
C20:2	-	-	-
C20:3	-	0.14 ± 0.02 ^A^	-
C20:5	-	1.06 ± 0.04 ^A^	-
C21:0	-	0.28 ± 0.02 ^A^	-
C22:0	1.25 ± 0.04 ^A^	0.25 ± 0.03 ^B^	-
C22:1	0.48 ± 0.02 ^A^	0.35 ± 0.04 ^B^	0.11 ± 0.004 ^B^
C22:2	-	-	1.30 ± 0.09 ^A^
C23:0	-	-	-
C24:0	-	-	-
C24:1	2.53 ± 0.02 ^A^	0.19 ± 0.003 ^C^	0.33 ± 0.08 ^B^
SFA	22.04 ± 0.27 ^A^	31.64 ± 0.69 ^A^	20.7 ± 0.25 ^C^
MUFA	69.52 ± 0.34 ^A^	50.54 ± 0.64 ^C^	65.04 ± 0.48 ^B^
PUFA	8.44 ± 0.21 ^C^	16.15 ± 0.43 ^A^	14.25 ± 0.38 ^B^
TUFA	77.96 ± 0.55 ^A^	66.69 ± 0.68 ^B^	79.29 ± 0.9 ^A^

Values are means ± standard deviations of three (*n* = 3) measurements. Different superscript uppercase letters in the same rows indicate significant differences (*p* < 0.05).

**Table 2:** Fatty acid composition (%) in Hass avocado pulp subjected to various drying methods.

		Pulp	
Fatty Acid	HAD	VD	HAMD
C14:0	0.08 ± 0.005 ^B^	0.10 ± 0.004 ^A^	0.02 ± 0.001 ^C^
C16:0	26.15 ± 0.48 ^B^	30.18 ± 0.51 ^A^	26.01 ± 0.35 ^B^
C16:1	12.23 ± 0.32 ^B^	12.11 ± 0.38 ^B^	13.02 ± 0.41 ^A^
C17:0	-	-	0.47 ± 0.02 ^A^
C17:1	-	-	0.15 ± 0.01 ^A^
C18:0	0.6 ± 0.03 ^B^	0.69 ± 0.05 ^A^	0.60 ± 0.06 ^A^
C18:1	44.42 ± 0.94 ^A^	42.81 ± 0.47 ^B^	44.01 ± 1.06 ^A^
C18:2	15.44 ± 0.61 ^A^	10.19 ± 0.23 ^C^	13.95 ± 0.33 ^B^
C18:3	0.76 ± 0.01 ^A^	0.57 ± 0.02 ^B^	0.79 ± 0.04 ^A^
C20:0	0.08 ± 0.01 ^B^	0.19 ± 0.02 ^A^	-
C20:1	0.13 ± 0.01 ^B^	0.16 ± 0.01 ^A^	-
C20:2	-	-	-
C20:3	-	-	-
C20:5	-	1.15 ± 0.06 ^A^	-
C21:0	-	-	-
C22:0	-	0.08 ± 0.01 ^A^	-
C22:1	-	0.38 ± 0.04 ^A^	-
C22:2	-	-	0.54 ± 0.02 ^A^
C23:0	-	0.19 ± 0.006 ^A^	-
C24:0	-	-	0.26 ± 0.007 ^A^
C24:1	-	1.00 ± 0.02 ^A^	-
SFA	26.91 ± 0.46 ^B^	31.43 ± 0.48 ^A^	26.89 ± 0.42 ^B^
MUFA	56.78 ± 0.98 ^A^	56.46 ± 0.65 ^A^	57.14 ± 1.01 ^A^
PUFA	16.20 ± 0.56 ^A^	11.91 ± 0.25 ^C^	15.28 ± 0.48 ^B^
TUFA	72.98 ± 1.43 ^A^	68.37 ± 0.82 ^B^	72.42 ± 1.45 ^A^

Values are means ± standard deviations of three (*n* = 3) measurements. Different superscript uppercase letters in the same rows indicate significant differences (*p* < 0.05) among drying methods (HAD—hot-air drying, VD—vacuum drying, HAMD—hot-air microwave drying).

**Table 3:** Fatty acid composition (%) in Hass avocado seed subjected to various drying methods.

		Seed	
Fatty Acid	HAD	VD	HAMD
C14:0	-	0.48 ± 0.02 ^B^	1.19 ± 0.04 ^A^
C16:0	15.19 ± 0.31 ^C^	19.27 ± 0.27 ^B^	24.77 ± 0.34 ^A^
C16:1	3.87 ± 0.12 ^C^	4.85 ± 0.11 ^B^	11.06 ± 0.14 ^A^
C17:0	-	-	0.63 ± 0.02 ^A^
C17:1	-	-	0.21 ± 0.006 ^A^
C18:0	0.62 ± 0.02 ^C^	0.82 ± 0.02 ^B^	1.46 ± 0.02 ^A^
C18:1	34.42 ± 1.04 ^B^	33.56 ± 0.71 ^B^	42.33 ± 0.60 ^A^
C18:2	42.45 ± 0.81 ^A^	39.83 ± 0.73 ^B^	13.67 ± 0.38 ^C^
C18:3	0.68 ± 0.04 ^B^	-	0.99 ± 0.11 ^A^
C20:0	0.63 ± 0.02 ^A^	-	-
C20:1	1.08 ± 0.02 ^A^	-	-
C20:2	-	-	-
C20:3	-	-	-
C20:5	-	-	-
C21:0	1.07 ± 0.04 ^A^	-	-
C22:0	-	-	-
C22:1	-	-	0.59 ± 0.02 ^A^
C22:2	-	-	1.18 ± 0.03 ^A^
C23:0	-	-	-
C24:0	-	-	0.76 ± 0.02 ^A^
C24:1	-	-	-
SFA	17.51 ± 0.39 ^C^	20.57 ± 0.31 ^B^	28.81 ± 0.44 ^A^
MUFA	39.37 ± 1.18 ^B^	38.41 ± 0.82 ^B^	54.19 ± 0.77 ^A^
PUFA	43.13 ± 0.85 ^A^	39.83 ± 0.73 ^B^	15.84 ± 0.41 ^C^
TUFA	82.50 ± 2.03 ^A^	78.24 ± 1.55 ^B^	70.03 ± 1.18 ^C^

Values are means ± standard deviations of three (*n* = 3) measurements. Different superscript uppercase letters in the same rows indicate significant differences (*p* < 0.05) among drying methods (HAD—hot-air drying, VD—vacuum drying, HAMD—hot-air microwave drying).

**Table 4:** Fatty acid composition (%) in Hass avocado peel subjected to various drying methods.

		Peel	
Fatty Acid	HAD	VD	HAMD
C14:0	-	-	0.20 ± 0.01 ^A^
C16:0	21.78 ± 0.24 ^B^	25.59 ± 0.25 ^A^	21.88 ± 0.79 ^B^
C16:1	9.96 ± 0.11 ^B^	10.68 ± 0.22 ^A^	10.71 ± 0.53 ^A^
C17:0	-	-	-
C17:1	-	-	0.13 ± 0.01 ^A^
C18:0	0.78 ± 0.02 ^A^	0.72 ± 0.02 ^B^	0.63 ± 0.03 ^C^
C18:1	47.47 ± 0.34 ^A^	44.53 ± 0.66 ^B^	41.71 ± 0.89 ^C^
C18:2	17.99 ± 0.29 ^A^	16.12 ± 0.43 ^B^	17.49 ± 0.64 ^A^
C18:3	1.72 ± 0.09 ^C^	2.10 ± 0.08 ^B^	3.08 ± 0.05 ^A^
C20:0	-	-	0.17 ± 0.02 ^A^
C20:1	-	-	0.16 ± 0.01 ^A^
C20:2	0.11 ± 0.01 ^A^	-	-
C20:3	-	-	-
C20:5	-	-	0.07 ± 0.01 ^A^
C21:0	-	-	-
C22:0	-	-	0.25 ± 0.01 ^A^
C22:1	-	-	-
C22:2	0.19 ± 0.01 ^C^	0.26 ± 0.01 ^B^	2.67 ± 0.07 ^A^
C23:0	-	-	0.04 ± 0.01 ^A^
C24:0	-	-	0.32 ± 0.01 ^A^
C24:1	-	-	0.12 ± 0.01 ^A^
SFA	22.56 ± 0.28 ^C^	26.31 ± 0.26 ^A^	23.49 ± 0.8 ^B^
MUFA	57.43 ± 0.46 ^A^	55.21 ± 0.65 ^B^	52.83 ± 0.98 ^C^
PUFA	20.01 ± 0.3 ^B^	18.48 ± 0.4 ^C^	23.31 ± 0.56 ^A^
TUFA	77.44 ± 0.73 ^A^	73.69 ± 0.99 ^B^	76.14 ± 1.34 ^A^

Values are means ± standard deviations of three (*n* = 3) measurements. Different superscript uppercase letters in the same rows indicate significant differences (*p* < 0.05) among drying methods (HAD—hot-air drying, VD—vacuum drying, HAMD—hot-air microwave drying).

**Table 5:** Functional indicators of dried parts of avocado (Hass cultivar).

		TUFA/SFA	PUFA/SFA	H/H Ratio	IA
Pulp	Fresh	3.54	0.38	3.26	0.26
	HAD	2.71	0.60	2.31	0.36
	VD	2.17	0.38	1.77	0.45
	HAMD	2.69	0.57	2.28	0.36
Seed	Fresh	2.11	0.51	1.93	0.46
	HAD	4.71	2.46	4.91	0.19
	VD	3.80	1.94	3.72	0.25
	HAMD	2.43	0.55	2.24	0.38
Peel	Fresh	3.83	0.69	3.55	0.26
	HAD	3.43	0.89	3.10	0.28
	VD	2.80	0.70	2.46	0.35
	HAMD	3.24	0.99	2.94	0.30

**Table 6:** Tocopherols content (µg/g of dry weight) in fresh avocado parts (peel, pulp, seed).

	α-Tocopherol	γ-Tocopherol	δ-Tocopherol	Total Tocopherols	Vitamin E Activity
Peel	57.57 ± 1.18 ^a^	43.23 ± 1.04 ^a^	82.42 ± 1.55 ^a^	183.2 ± 3.77 ^a^	62.72 ± 1.30 ^a^
Pulp	24.57 ± 1.01 ^b^	26.26 ± 0.77 ^b^	6.57 ± 0.29 ^b^	57.41 ± 2.07 ^b^	27.26 ± 1.08 ^b^
Seed	13.61 ± 0.32 ^c^	-	-	13.61 ± 0.32 ^c^	13.61 ± 0.32 ^c^

Values are means ± standard deviations of three (*n* = 3) measurements. Different superscript lowercase letters in the same column indicate significant differences (*p* < 0.05) among different avocado parts (pulp, seed, peel).

**Table 7:** Tocopherols content (µg/g of dry weight) in avocado peel treated with different drying methods.

	α-Tocopherol	γ-Tocopherol	δ-Tocopherol	Total Tocopherols	Vitamin E Activity
HAD	69.53 ± 0.91 ^c^	55.13 ± 1.00 ^b^	129.14 ± 0.76 ^a^	253.8 ± 3.43 ^b^	76.33 ± 1.01 ^c^
VD	108.81 ± 1.48 ^a^	64.80 ± 0.94 ^a^	83.01 ± 0.60 ^b^	256.62 ± 3.02 ^a^	116.12 ± 1.54 ^a^
HAMD	91.11 ± 1.91 ^b^	47.10 ± 0.33 ^c^	130.24 ± 1.10 ^a^	268.45 ± 3.23 ^a^	97.12 ± 2.05 ^b^

Values are means ± standard deviations of three (*n* = 3) measurements. Different superscript lowercase letters in the same column indicate significant differences (*p* < 0.05) between drying methods (HAD—hot-air drying, VD—vacuum drying, HAMD—hot-air microwave drying).

## Data Availability

The original contributions presented in the study are included in the article, further inquiries can be directed to the corresponding author.
